# The two halves of U-shaped mortality

**DOI:** 10.3389/fgene.2013.00031

**Published:** 2013-03-19

**Authors:** Daniel A. Levitis, Daniel E. Martínez

**Affiliations:** ^1^Max-Planck Odense Center on the Biodemography of AgingOdense, Denmark; ^2^Institute of Biology, University of Southern DenmarkOdense, Denmark; ^3^Department of Biology, Pomona CollegeClaremont, CA, USA

**Keywords:** evolutionary demography, life-history, mortality patterns, ontogenescence, senescence

## Abstract

Gerontology focuses on deterioration with increasing age, but in most populations most variables, including survival probability, improve at early ages (ontogenescence) before deteriorating at advanced ages (senescence). The extent to which gerontology needs to consider this U-shaped pattern of risk over age depends upon the mechanistic, demographic and evolutionary links and interactions between ontogenescence and senescence. In reading the literature on both senescence and ontogenescence, and in interacting with other biogerontologists, we have encountered a set of what we view as inaccurate or oversimplified claims about ontogenescence, its relationship to senescence and its importance to gerontology. Here, after briefly introducing ontogenescence, we address four of these claims. We demonstrate the counterfactual nature of **Claim 1.** Ontogenescence is an environmental effect largely absent in protected environments. We then briefly review the literature which leads to **Claim 2. **Senescence and ontogenescence are parts of the same phenomenon, and describe why we reject this view. We then explain why the rejection of Claim 2 does not necessarily support **Claim 3**, the idea that senescence and ontogenescence are easily separable. Finally, we examine **Claim 4.** Gerontologists don’t need to think about ontogenescence, and give some examples of why we consider this misguided.

## INTRODUCTION

In aging research, we tend to think of variables changing in one direction with increasing age, from better to worse. But between birth and old age the picture is more complex; a wide range of variables first improve, then deteriorate. This is true of cellular (Stolzing et al., 2008), psychological (Williams et al., 2005), physiological (Choi et al., 2011), mechanical (Bohannon et al., 2006), and economic (Bucks et al., 2009) variables, among others. Things tend to get better before they get worse.

Given this pattern, it may be unsurprising that age-specific mortality is U-shaped for a wide range of organisms. Risk of death is high early in life, declines rapidly during ontogenesis, bottoms out around the age of maturity and then increases exponentially through advanced age. The details of the decline (labeled ontogenescence by Levitis, 2011) and increase (senescence) of mortality risk are variable, but both are very widely observed, to the point that U-shaped mortality is a reasonable default hypothesis for most biological populations.

There is no general consensus on the relationship between senescence and ontogenescence. Ecologists have generally treated them as separate, studying mortality at the beginning of life in one set of studies (e.g., Gosselin and Qian, 1997), and mortality late in life in another set (e.g., Monaghan et al., 2008). Gerontologists focus on aging and usually take neither data on, nor interest in, the periods before adulthood. Theorists in evolutionary demography, have tended to either ignore ontogenescence (e.g., Medawar, 1952; Jones et al., 2008) or treat ontogenescence and senescence as parts of a single process (e.g., Hamilton, 1966; Lee, 2003; Robson and Kaplan, 2003). Each of these approaches has its merits, but none is based on a broad view of the relationship between senescence and ontogenescence. Below we address what we see as common, but false or oversimplified, claims in aging research regarding the nature of ontogenescence, its relationship to senescence and its importance to biogerontologists.

## CLAIM 1: ONTOGENESCENCE IS AN ENVIRONMENTAL EFFECT LARGELY ABSENT IN PROTECTED ENVIRONMENTS

Environment has a strong effect on mortality risk. While environmental effects can be observed at all ages – a Japanese woman reaching her 100th birthday had a 27.2% chance of dying in the following year in 2009 compared to a 54.2% chance for similar centenarians in 1949 (Human Mortality Database, 2011) – the most pronounced improvements in survival observed in humans with improving environments have been in infants and children. Indeed, a healthy child born to parents in a rich industrialized nation now has an excellent chance of surviving to adulthood – 99.5% in Japan in 2009 vs. 82.6% in 1949 (Human Mortality Database, 2011). We have frequently encountered the view among gerontologists, based on this remarkable decrease in the level of early mortality, that it is established fact that ontogenescence is mostly or entirely an environmental effect. Rather than stating this view in print, those holding it generally ignore pre-adult mortality entirely.

Two lines of evidence disprove this claim. First, ontogenescence is still clearly observable in the mortality patterns of even the richest and most industrialized nations. This is true of mortality between birth and age 12, and between conception and birth. For example, Macklon et al. (2002) estimate that in modern rich nations, 60% of pregnancies are subclinical losses (the mother experiences no symptoms of pregnancy and does not miss a cycle), with a further 10% lost with symptoms, leading to 30% survival to birth. While estimates vary (Goldstein, 1994; Wilcox et al., 1988, 1999; Holman and Wood, 2001; Macklon et al., 2002) on risk of a human conceptus spontaneously aborting in its first month, it is on the same order of magnitude as the risk of a 110 year old dying in the next year, 50% (Gampe, 2010). While environment clearly has a strong effect on the level of early life mortality, losses at the beginning of life are considerable for all studied human populations, and the slope of mortality over age, by which ontogenescence is defined, seems almost always to decline.

Second, ontogenescence is too broadly observed a phenomenon to explain in purely environmental terms. For no vertebrate population in any environment has mortality from conception to maturity been tracked and not found to generally decline with development. This was first proposed as being true of all natural mammal populations (Barlow and Boveng, 1991; Caughley, 1966; Loison et al., 1999), but has been documented in a wide array of other animals (Jones et al., 2008; Powell, 2009; Scheuerlein et al., 2012), and plants (Harcombe, 1987). More broadly still, for every phylum of living things for which we have the necessary relevant data, there are examples of ontogenescence (Levitis, 2011). U-shaped mortality is also observable when one considers individual causes of death (Kruger and Nesse, 2004), for example when the young and old are most susceptible to predation (DelGiudice et al., 2002). Ontogenescence occurs across taxa, life-histories, causes of death, and environments, requiring explanations rooted in the basic biology shared by all living things. The hypotheses potentially explaining this breadth are reviewed in Levitis (2011). In short, there are good biological reasons to expect ontogenescence to be observed across environments, because ontogenescence is driven by biological factors such as genetic and stochastic heterogeneity and the complexity of the developmental program. The susceptible, untested young must navigate the series of dangerous transitions which together constitute ontogenesis, and many of them don’t make it.

## CLAIM 2: SENESCENCE AND ONTOGENESCENCE ARE PARTS OF THE SAME PHENOMENON

The fact that both ontogenescence and senescence occur so broadly, and often occur together, has led influential theorists to propose that they may be parts of the same phenomenon. Caughley (1966) was perhaps the first to note that U-shaped mortality is widely conserved across taxa (in this case mammals) and attempt to explain that pattern. He speculated that this may be due to the inverse U-shape of Fisher (1930) reproductive value (mean expectation of future reproduction given age-specific fecundity and death rates) over age. In the same year, Hamilton (1966) showed that the selective reasoning behind Caughley’s suggestion was flawed; the force of selection against mortality at each age (which Caughley did not consider), rather than reproductive value (which he did), should determine the accumulation of mutations driving age-specific risk. Unlike reproductive value, the force of selection against mortality should generally be constant until reproduction begins, and as such the shape of reproductive value should not lead to ontogenescence.

Hamilton offered a different explanation for the U; he speculated that the force of selection against mortality may be U-shaped under special circumstances, specifically in those cases where dying earlier rather than later in development imposes a smaller cost on the individual’s kin. The individual, or more plausibly its kin, may recognize that resources would be better invested in producing a sibling, replacing rather than maintaining a suboptimal offspring. Lee and colleagues (Lee, 2003, 2008; Chu et al., 2007), influenced by Robson and Kaplan (2003) have modified and built from Hamilton, using economic theory and kin selection to predict U-shaped mortality in humans based on the pattern of intergenerational transfers of caloric resources in hunter-gatherer groups. The economic/evolutionary mechanisms they propose likely function to modify the details of the U in humans, but cannot be seen as a general explanation for this pattern which also occurs in a wide range of organisms lacking the kin sharing on which their hypothesis is based. Hamilton’s prediction that we should not see ontogenescence in cases where infant mortality has little effect on surviving kin has not panned out. Many excellent examples of this can be found in the literature on marine invertebrate larvae (Rumrill, 1990; Morgan, 1995) and the question is treated in more detail in Levitis (2011). Nevertheless, Lee’s formulation of Hamilton’s sibling replacement hypothesis continues to influence the thinking of many evolutionary demographers (e.g., Hawkes, 2010; Burger et al., 2012; Gurven et al., 2012).

More broadly applicable hypotheses treat ontogenescence and senescence as separate if related phenomena. For example, Moorad and Promislow (2008) apply theory from quantitative and evolutionary genetics to show that if phenotypic complexity declines with age, and if the force of selection against mortality decreases with age, U-shaped mortality can be predicted based on a single model. They are vague on the type of complexity to which they refer, and why it should decline, but some types of complexity, such as the complexity of the changes occurring in gene expression at each stage of development, may indeed decline with age (Levitis, 2011). In their model, rapidly declining complexity during ontogenesis leads to ontogenescence, while the more slowly declining force of selection leads to senescence. Similarly, many life-course optimization models (Vaupel et al., 2004; Munch and Mangel, 2006; Chu et al., 2007; Baudisch, 2008) show U-shaped mortality, but ontogenescence is driven by increasing size and robustness while senescence is caused by damage accumulation or decreasing selective value. The evolutionary phenomena causing the two halves of the U within these models are distinct. Our view, described in greater detail in Levitis (2011) is that ontogenescence, like senescence, arises from constraints on what natural selection can accomplish. But where the evolutionary roots of senescence are to be found in the diminishing selective relevance of traits expressed at higher ages, most traits which decrease the chance of dying before reaching reproductive age should be strongly selected for. That ontogenescent mortality remains so high in such a broad range of organisms suggests to us that far from resulting from kin selection, or being part of an optimal life-cycle, ontogenescence is primarily the result of mechanistic constraints not generally included in evolutionary theoretical models.

**FIGURE 1:**
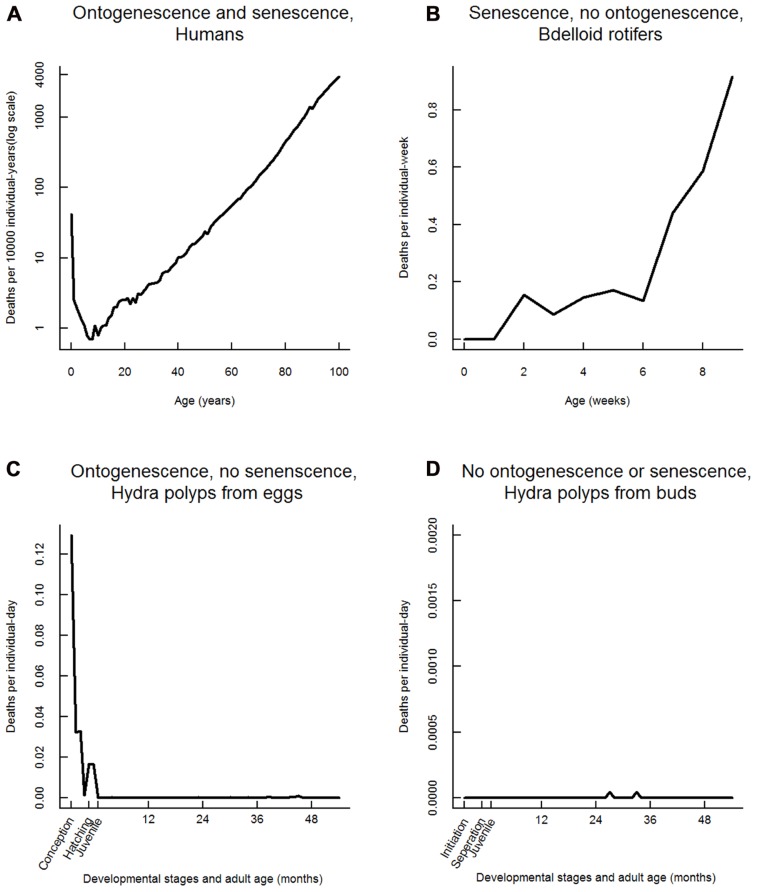
**Age-specific survival for four populations, illustrating the separability of ontogenescence from senescence**. **(A)** Humans (United Kingdom period data for 2009 shown) experience classical U-shaped mortality, with both ontogenescence and senescence (Human Mortality Database, 2011). **(B)** Bdelloid rotifers (*Macrotrachela quadricornifera*) in the Ricci lab avoid ontogenescence, but not senescence (Ricci et al., 2007 and personal communication, Ricci, 2011). Data from 24 individuals are shown, but this pattern is seen repeatedly across trials and bdelloid species. **(C)** Hydra of strain AEP developing in and hatching from eggs suffer intense ontogenescence (159/344 pre-adults died, meaning 46% mortality between egg formation and reproductive maturity), but do not senesce. **(D)** Hydra of the same strain, under the same conditions, but developing as buds rather than eggs, experience no age-related mortality (0/122 pre-adults died) as juveniles or adults.

If senescence and ontogenescence always co-occurred, as they do in humans (**Figure 1A**) we could still question whether they are causally linked, given the fact that senescence and ontogenescence are among the most widespread of life-history traits. However, given that we now have examples of populations which senesce but do not ontogenese, and those which ontogenese but do not senesce, we argue that separate explanations are needed.

An example of a population which senesces without ontogenescence can be found in some laboratory populations of *Caenorhabditis elegans* kept under carefully controlled circumstances (Wiegant et al., 2009). Similarly, bdelloid rotifers senesce but can avoid ontogenescence (**Figure 1B**). While senescence is apparent in all cohorts in the lab, 100% survival from egg initiation to adulthood can be achieved with careful husbandry (Ricci et al., 1987; Santo et al., 2001). The fact that ontogenescence is not seen under these conditions, but is observed under other conditions – e.g., when eggs are dehydrated then rehydrated (Ricci, 1998) – highlights the fact that environment can induce ontogenescence. *Hydra oligactis* which normally does not senesce, but rapidly deteriorates after sexual reproduction may be a case of environmentally induced senescence (Yoshida et al., 2006; Martínez and Bridge, 2012). Indeed, the ultimate goal of many biogerontologists is to learn how to induce the lack of senescence in humans.

Which ontogenescing populations lack senescence? Ontogenescence is broadly observed in reptiles, yet for very few species of reptile has somatic senescence been demonstrated (Bronikowski, 2008; Massot et al., 2011) and some ontogenescing reptiles, such as the desert tortoise, *Gopherus agassizii*, show negative senescence long into adulthood (Turner et al., 1987), based on increasing size (a component of ontogenescence). We do not know if tortoises kept in a safe environment where size loses its protective effect would show a very different pattern of aging. Thus we do not know if this species has senescence in terms of inherent mortality risk, as it could be masked by ontogenescence in this species.

Another very revealing example may be found in the laboratory, in the genus Hydra. Martínez (1998) has shown that *H. vulgaris* cohorts show no signs of senescence over several years. Studying *H. vulgaris* strain AEP (methods follow Martínez, 1998), which reproduce readily through both budding and egg production, we have found that offspring produced as buds show no ontogenescence while strong ontogenescence is detectable in offspring developing in and hatching out of eggs. However, individuals produced either way do not senesce, indicating that ontogenescence can be triggered without inducing senescence (**Figures 1C,D**).

The prevalence of U-shaped age-specific mortality is likely due to the extreme prevalence of the two phenomena which overlap to form the U. Efforts to explain this broadly occurring pattern based on the characteristics of a single taxon are likely to remain unsatisfying, and efforts to explain both phenomena based on a single mechanism are likely to remain unsuccessful.

## CLAIM 3: SENESCENCE AND ONTOGENESCENCE ARE EASILY SEPARABLE

It is tempting to assume that phenomena with different ultimate causes can be easily disentangled during data analysis, but this is not always so. The measurement of senescence and ontogenescence is complicated by the fact that the demographic mechanisms underlying each are not strictly separated by age. For example, ontogenescence may be driven by a pattern of demographic selection in a population with heterogeneous frailties (Vaupel and Yashin, 1985), but this same process may slow or even halt the appearance of senescence on a population level (Vaupel et al., 1998). Estimation of the exponential rate of aging of a population (Gompertz’ *b*), is considerably complicated by such heterogeneity effects, but also by the need to estimate and subtract out non-senescent mortality (Gage, 1988). As in Moorad and Promislow’s (2008) model, U-shaped mortality may best be understood as the multiplication, rather than addition, of causally distinct but interacting ontogenescent and senescent mechanisms, considerably complicating attempts to decompose mortality into senescent and ontogenescent components. Indeed, the very question of at what age senescent mortality begins is extraordinarily difficult to answer in part because ontogenescent mortality would swamp out any signal of the Gompertizian pattern early in life. The question is not trivial, as damage-accumulation based theories of aging predict that senescence should start at or before birth (Abrams, 1991; Milne, 2006), while Hamiltonian evolutionary theory predicts that senescence should start at the onset of reproduction (Hamilton, 1966), and demographic definitions suggest that senescence starts when mortality rate stops declining and starts increasing (Promislow, 1991; Milne, 2006). As a final example, consider the question of whether cancer deaths should be considered as senescent mortality. The incidence of cancer (all types pooled) is high very early in life (Young et al., 1986), drops off rapidly, and then rises through adulthood before leveling off and declining again at advanced age (Harding et al., 2011). This complexity may arise from the fact that age-specific cancer risk is an interaction between mechanisms of senescence, such as damage accumulation over age, and mechanisms of ontogenescence, such as growth, selective removal of the genetically frail and the dangers associated with biological transitions. In other words, the mechanisms underlying ontogenescence and senescence can interact in ways that make partitioning deaths into senescent and ontogenescent categories both statistically and conceptually difficult.

## CLAIM 4: GERONTOLOGISTS DON’T NEED TO THINK ABOUT ONTOGENESCENCE

Interactions between senescence and ontogenescence lead to the need for gerontologists to think about ontogenescence. In addition to the biological interactions, early and late mortality patterns interact at the population levels. Disease early in life can kill some offspring but also damage the survivors, leading to increased adult mortality. The opposite effect is also possible: disease during ontogenesis can selectively remove the frailest individuals, leaving a more robust population, or induce acquired immunity, thereby increasing adult life-expectancy. Where survival or health of offspring depends on the survival or health of adults, senescence may increase ontogenescent mortality. This in turn is thought to make the evolution of post-fertile survival – as described in the Grandmother (Hawkes, 2004) and Mother (Williams, 1957) Hypotheses – dependent upon the rate at which the young grow and become independent. The easing of population pressure caused by high juvenile mortality can affect adult survival and fertility, altering selective pressure for longevity. More generally, natural selection will tend to optimize the set of vital rates as a whole, meaning that ontogenescent and senescent mortality patterns will influence each other’s evolution through life-history tradeoffs. This optimization occurs within the constraint imposed by biological mechanisms, such that selection for traits at one end of the lifespan can alter the mortality pattern at both ends of the U. Indeed, much of the evolutionary theory of aging relies on such agonistic pleiotropies (Williams, 1957) and the relatively few examples when the benefits and costs are well known both early and late in life (e.g., Vermeulen and Bijlsma, 2006) are of great importance in understanding the mechanisms behind the evolution of senescence. A great many “aging genes” may well be developmentally important alleles with pleiotropic effects (Promislow, 2004); understanding these pleiotropies is necessary if we are to put aging-focused systems biology into evolutionary context or practical use.

On a more prosaic level, forgetting that things get better before they get worse can lead to easily avoidable mistakes in data analysis. We have encountered several published figures showing a monotonic line fitted through a scatter plot that clearly shows a pattern of first fall, then rise (e.g., Arking et al., 2002, Figure 4c), or rise, then fall over age. The usual solution to this problem, to exclude all data during the ages when things improve, disregards vital information and biases the analysis by allowing the researcher to choose those data that show the expected pattern. In deciding if something is a primary cause, secondary cause or symptom of senescence, it is useful to know if its pattern of change is constant across ages or if deterioration is seen only during those ages when symptoms of senescence are evident. This requires us to know something about the early stages of life.

As a final example, Sun et al. (2009) show that diet early in life (caloric or protein restriction) can have a significant effect on longevity. Their analysis demonstrates that closer attention to the events of early life can be important to the interpretation of longevity data. Their interpretation, that dietary restriction is leading to slower aging, is possible. An alternative interpretation, that aging is starting later because development takes longer, should also be considered. Distinguishing between these two, by carefully observing development and the use of advanced demographic data analysis, would tell us a great deal about both the onset and process of senescence.

## CONCLUSION

While ontogenescence and senescence may both be characterized as side-effects of evolution (Medawar, 1952; Levitis, 2011), they are separate in ultimate causation, and therefore gerontologists are justified in seeing ontogenescence as distinct from their area of interest. However, because ontogenescence interacts with senescence in the mechanisms, measurement, ecology, and evolution of age-specific mortality, drawing a clear line between senescence and ontogenescence is difficult. Ontogenescent mortality, like senescence mortality, is likely to continue to be reduced by improving technology, but neither will be completely eliminated any time soon, and our underlying biology will remain one that evolved in the context of individual improvement, then decline, over age. Gerontology would be well served by an understanding of ontogenescence and how it influences the pattern of senescence.

## HYDRA LAB METHODS

Hydra pre-adult mortality data on 122 buds and 344 eggs/hatchlings were gathered by daily checking stage and survival of offspring. Hydra adult mortality was assessed by thrice weekly checks for 6 years on 150 adults produced from buds, and 120 from eggs. Hydra were kept individually in well plates, fed 9 *Artemia* nauplii per individual per week and water was changed three times per week. Culture methods are described in detail in Martínez (1998).

## Conflict of Interest Statement

The authors declare that the research was conducted in the absence of any commercial or financial relationships that could be construed as a potential conflict of interest.
